# Fast and Sensitive Method for Determination of Domoic Acid in Mussel Tissue

**DOI:** 10.1155/2016/8404092

**Published:** 2016-01-21

**Authors:** Elena Barbaro, Roberta Zangrando, Carlo Barbante, Andrea Gambaro

**Affiliations:** ^1^Department of Environmental Sciences, Informatics and Statistics, Ca' Foscari University of Venice, Via Torino 155, Mestre, 30170 Venice, Italy; ^2^Institute for the Dynamics of Environmental Processes, CNR, Via Torino 155, Mestre, 30170 Venice, Italy

## Abstract

Domoic acid (DA), a neurotoxic amino acid produced by diatoms, is the main cause of amnesic shellfish poisoning (ASP). In this work, we propose a very simple and fast analytical method to determine DA in mussel tissue. The method consists of two consecutive extractions and requires no purification steps, due to a reduction of the extraction of the interfering species and the application of very sensitive and selective HILIC-MS/MS method. The procedural method was validated through the estimation of trueness, extract yield, precision, detection, and quantification limits of analytical method. The sample preparation was also evaluated through qualitative and quantitative evaluations of the matrix effect. These evaluations were conducted both on the DA-free matrix spiked with known DA concentration and on the reference certified material (RCM). We developed a very selective LC-MS/MS method with a very low value of method detection limit (9 ng g^−1^) without cleanup steps.

## 1. Introduction

Domoic acid (DA) is a neurotoxic amino acid produced by different algae, including, principally from* Pseudo*-*nitzschia*, pennate diatoms. Due to their filter feeding nature, bivalve mollusks can accumulate high concentration of many contaminants and, during the algal bloom, the accumulation of domoic acid is the main cause of amnesic shellfish poisoning (ASP) [1]. The toxicity of DA is caused by its chemical structure, which is very similar to that of two neurotransmitter amino acids, L-glutamic acid and kainic acid [2]. After ingestion, DA has an effect on the central nervous system because it has a higher affinity with the receptors than do glutamic acid and kainic acid, causing depolarisation of the neurons [3].

Several countries promote monitoring programs to ensure consumer protection and the total concentration of DA must not exceed 20 *μ*g per gram of wet tissue [4]. However, the study of uptake, distribution, transformation, and elimination of ASP phenomena requires very sensitive and selective analytical methods for DA determination.

The detection of this toxin in mussels can be conducted through either biochemical assay or the instrumental method [5]. A mouse assay developed for the identification of PSP (paralytic shellfish poisoning) toxins was proposed by the Association of Official Analytical Chemists (AOAC) also for DA detection. The mussel tissue is extracted with an acid solution (0.1 N of hydrochloric acid), after which the extract is injected into the mouse. This method is not useful for regulatory purposes because the first symptoms begin with a DA concentration of 40 *μ*g g^−1^.

The instrumental methods are the most sensitive techniques to determine DA and liquid chromatography coupled with ultraviolet detection (LC-UV) is one of the detection methods suggested to determine DA in the mussels [6]. The mussel tissues can be extracted using the AOAC procedure [7] or a methanol aqueous solution (1 : 1), which achieved the best recovery and extract stability [6]. The method detection limits ranged between 0.1 and 1 *μ*g of DA for gram of tissue, depending on the sensitivity of the UV detector. The main disadvantage is the presence of several interferences, which can introduce false positives. A purification procedure with solid phase extraction (SPE) was therefore necessary to reduce the errors [6, 7].

The capillary electrophoresis coupled with UV detector is another technique commonly used for DA determination in mussels, but it requires two purification phases with anionic and cationic SPEs [8]. The sensitivity can be increased by using a fluorimetric detector with 4-fluoro-7-nitro-2,1,3-benzoxadiazole as derivatization reagent. This method permits obtaining a detection limit of 6 ng of DA per gram tissue [9].

Liquid chromatography coupled with mass spectrometry (LC-MS) guarantees the best sensitivity but also a great selectivity on the determination of DA in the mussel tissue [5, 10–12]. Hess et al. [13, 14] compared LC-MS and LC-UV, underlining that the latter technique could yield a false positive when the samples contain interfering species. In the literature, one of the main problems in the DA determination of mussel samples is the matrix effect and a labor-intensive anion exchange solid phase extraction is usually carried out for an efficient sample cleanup [13, 14]. Regueiro et al. [15] developed an online purification method coupled to LC-MS analysis, minimizing the matrix 2 effect and with a preconcentration of samples.

The aim of our study was to develop a simple and fast preanalytical procedure for the quantitative determination of DA in mussel tissue without purification phase. The study of extraction step permitted individuating the solvent that minimizes the matrix effect without a labor clean-up step. This approach will reduce the time of sample preparation, guaranteeing the selectivity and the sensitivity due to LC-MS/MS analysis. This is the first study that quantified DA in mussel tissue with internal standard in order to correct the response of the mass spectrometer for random fluctuations.

## 2. Experimental Section

### 2.1. Chemicals and Materials

DA (purity ≥ 98%) was purchased from Vinci Biochem (Florence, Italy), and leucine enkephalin (ENK) was bought from Sigma Aldrich (Steinheim, Germany).

Certified reference material CRM-ASP-MUS-D, containing a thermally sterilized homogenate of mussel tissue (*Mytilus edulis*) contaminated with DA with a concentration of 49 ± 3 *μ*g g^−1^, was purchased from the Canadian National Research Council.

The ultrapure water (18.2 MΩ cm, 0.01 TOC) was produced by means of a Purelab Ultra system, consisting of a Purelab Option R purification plant system coupled to Purelab Ultra Analytical ultrapure system (Elga, LabWater, High Wycombe, UK). HPLC/MS-grade methanol and acetonitrile were obtained from Romil Ltd. (Cambridge, UK). The mobile phase additive formic acid was purchased from Fluka (Sigma Aldrich, Buchs, Switzerland). The ZIC-HILIC column (4.6 × 150 mm, 100 Å) was produced from SeQuant (Umea, Sweden).

The mussel tissues were extracted by sonification in a polyethylene tube (15 mL, Iwaki) and then filtrated using syringe PTFE fiber filters (0.45 *μ*m, *ø* 25 mm, National Scientific Company, Rockwood, TN, USA).

### 2.2. Sample Processing and Analysis

The mussel samples were stored in aluminum at −20°C until extraction.

In order to guarantee the sample representativeness, one hundred animals were homogenized with agate mortar and pestle and about 5 g of tissue was treated with an Ultra-Turrax homogenizer system, previously cleaned with water and methanol.

A small portion (20 mg) was spiked with the internal standard ENK (200 ng absolute) into a polyethylene tube before being ultrasonically extracted with 10 mL of methanol for 15 minutes at ambient temperature. The extract was centrifuged at 3000 rpm for 5 minutes and then filtrated into a 50 mL conic flask through a 0.45 *μ*m PTFE filter. The pellet was extracted again with another 10 mL of methanol, centrifuged and filtrated into the same conic flask in order to gather two extracts.

**Figure 1:**
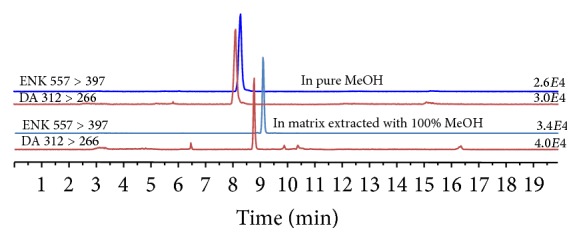
Comparison of extract ion chromatogram of DA and ENK standard solution diluted in pure methanol and in matrix extracted with methanol.

**Figure 2:**
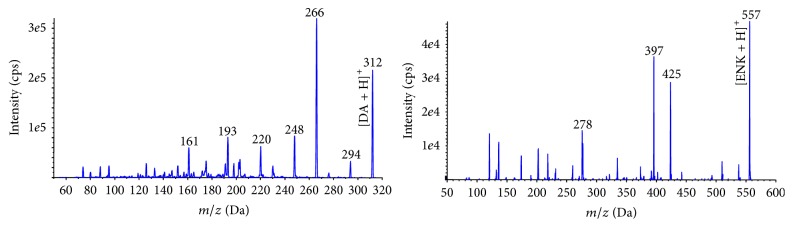
Product ion MS/MS spectra of DA and internal standard ENK in positive ion mode, using a collision energy of 10 eV.

An aliquot of this filtrate was analyzed using the instrumental method developed by Barbaro et al. [16]. Briefly, an Agilent 1100 Series HPLC System (Waldbronn, Germany) equipped with a binary pump, vacuum degasser, and autosampler was coupled with an ESI electrospray ion source and an API 4000 Triple Quadrupole Mass Spectrometer (Applied Biosystem/MDS SCIEX, Concord, Ontario, Canada). Chromatographic separation was performed using a 4.6 × 150 mm ZIC-HILIC column with mobile phase gradient elution consisting of water with 0.1% formic acid as eluent A and acetonitrile with 0.1% formic acid as eluent B. A binary elution gradient program at a flow rate of 0.5 mL min^−1^ was used as follows: 0–2 min, 85% eluent B; 2–5 min gradient from 85 to 15% eluent; 5–10 min, 15% eluent B; 10–12 min gradient to 85% eluent B; 12–20 min, 85% eluent B. The volume of sample injected for analysis was 100 *μ*L. The chromatographic separation for a standard solution of DA and ENK diluted in pure methanol is reported in Figure 1 (top chromatograms). The ESI ion source was operated in positive polarity during the DA analysis and the data were acquired in MRM mode, enabling highly selective and sensitive detection of selected fragments. The MRM transitions 312 > 266, 312 > 248, and 312 > 220 were used for DA while the transitions 557 > 397, 557 > 425, and 557 > 278 were employed for ENK. The most intense fragments, shown in the product ion mass spectra (Figure 2), were used for sample quantification while the other fragments were used to confirm the compound identity. The quantification was performed using ENK as internal standard in order to correct instrumental fluctuation. By adding the internal standard at the beginning of the extraction procedure, we could also correct DA losses during the sample preparation steps.

### 2.3. Quality Control

We used a series of standard solutions prepared in pure methanol and in DA-free matrix extracted with two different solvents (methanol and 80 : 20 methanol and pure water) at concentrations between 0.01 and 100 *μ*g L^−1^ with ENK as internal standard at a constant concentration of 10 *μ*g L^−1^. We have evaluated the linearity and reproducibility. We have also considered the stability of DA in methanol, obtaining good reproducibility (RSD below of 10%) in two weeks for our extracted samples. These solutions were also used to evaluate the matrix effect.

The analytical procedure to determine DA in mussel tissue samples was validated by estimating the trueness and repeatability of sample treatment process. We have used 20 mg of DA-free mussel tissue (*n* = 3) spiked with 1 *μ*g of DA and 0.2 *μ*g of internal standard ENK and 20 mg of certified reference material CRM-ASP-MUS-D (*n* = 3) spiked with 0.2 *μ*g of ENK. These samples were extracted as reported for the real samples in Section 2.2.

## 3. Results and Discussion

The main aim of this study was to develop a simple and fast procedure for sample preparation that did not require any long and expensive purification steps while also minimizing the matrix effect.

The most extensively used extraction procedures resort to 0.1 N hydrochloric acid or aqueous methanol (1 : 1) [6, 7]. However DA can degradate using low pH in only one week [6]. Extraction with water/methanol 1 : 1 guarantees a total recovery of DA, with also extraction of several interfering species [6].

In the literature [6, 7], the amount of mussel tissue was extracted in order to determine DA concentration is usually 1 gram. Instead, we proposed extracting 20 mg of mussel tissue (a small aliquot of 5 g of sample previously homogenized), in order to minimize the matrix effect in the ionization source during the MS instrumental analysis. This key change, combined with the highly sensitive LC-MS/MS method developed in our previous study [16], is to our knowledge the most sensitive and selective method to analyze DA in mussel tissue, as demonstrated below. The main aims of our development were to obtain an extract with low interfering species and to perform a method with high sensitivity and high selectivity but without a labor clean-up step.

For both purposes, we have considered an increment in the percentage of organic solvent in a mixture of water/methanol during the extraction in order to increase the precipitation of protein and to reduce the extraction of lipid substances which are the main interfering species, although the solubility of DA decreases from water (7.6 g L^−1^) to methanol (0.66 g L^−1^) [17]. Methanol and a mixture 80 : 20 methanol/water were used during the development of the preparative method to establish the best extraction solvent for DA determination without purification steps except for filtration with 0.2 *μ*m PTFE filter to remove the particulate before the analysis.

For both extraction solvents, we estimated the possible matrix effect and the recovery efficiency (RE) using a DA-free mussel matrix spiked with a known DA concentration.

The matrix effect consists in an enhancement or suppression of ion intensity in the HPLC-MS interface due to undetected matrix components which coelute with the target compound and its evaluation is heartily recommended by the Guidance for Industry on Bioanalytical Methods [18].

**Figure 3:**
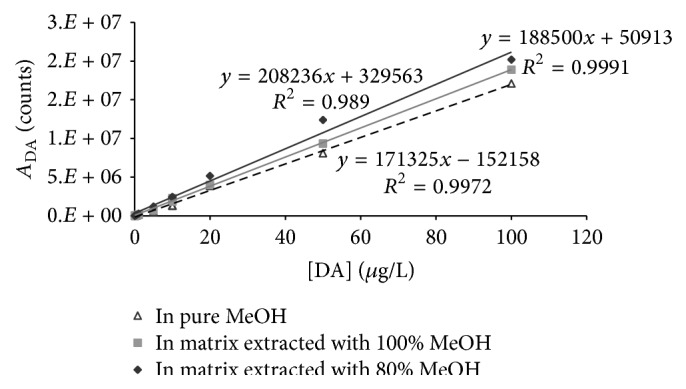
A qualitative evaluation of matrix effects in the method for the determination of DA in mussel tissue using two different extraction solvents, methanol/water 80 : 20 and methanol.

A qualitative estimation of the matrix effect for both extraction methods was carried out by comparing the calibration curve (Figure 3) with concentrations between 0.01 and 100 *μ*g L^−1^ in pure methanol and in the DA-free matrix after the extraction with both different considered solvents. Figure 2 shows that the matrix extracted with 80 : 20 methanol/water produced a greater enhancement of the positive ion intensity than the matrix extracted with only methanol, where the slope is very similar to that obtained by the synthetic calibration curve.

**Figure 4:**
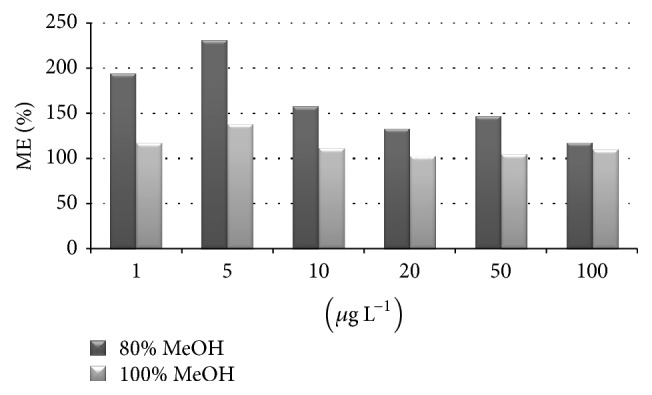
A quantitative estimation of matrix effect for the determination of DA in mussel tissue using two different extraction solvents, methanol/water 80 : 20 and methanol.

A quantitative evaluation of the matrix effect, as suggested by Matuszewski et al. and Constanzer [19], highlighted the advantage to use 100% methanol as extraction solvent. We calculated the values of the matrix effect (ME%) for concentrations between 1 and 100 *μ*g L^−1^ by dividing the signal of a standard prepared in the sample extract with the response of a standard diluted in a pure solvent. A value similar to 100% means that there is no matrix effect, while values above or below 100%, respectively, indicate a suppression or enhancement of signal in the ion source. As shown in Figure 4, ME values were very close to 100% when using methanol as extraction solvent, while the methanol/water 80 : 20 mixture led to a considerable enhancement of signal in the ESI source.

The RE was also evaluated by dividing the signal response in extracts spiked with standards before sample treatment by the signal response of the same extracts after the sample was treated. Finally, we also considered the process efficiency (PE) as the product of ME and RE [19]. These parameters were calculated with a DA concentration of 50 *μ*g L^−1^ (*n* = 3), similar to the concentration obtained during the extraction of CRM-ASP-MUS-D mussel tissue. RE values show that the methanol/water 80 : 20 mixture permitted extracting only 14% of the DA from the mussel tissue, while 85% of DA was obtained by using methanol as extraction solvent. The combination of the ME and the procedure performance generated a mean PE value of 94% while only 21% was obtained with the mixture water/methanol. This is the first study to fully evaluate the ME for determining DA in mussel tissue, and we demonstrated that methanol is the best solvent to totally extract DA while minimizing the matrix effect.

In order to correct the response of the mass spectrometer for random fluctuations, we performed a quantification using the internal standard. This is the first study to use an internal standard for the determination of DA in mussels. We used ENK, the same internal standard applied in our previous study of the determination of DA in seawater samples [16]. In Figure 1, we have reported the comparison between the chromatogram of 1 *μ*g L^−1^ of DA and ENK diluted in pure methanol and in the DA-free matrix extracted with methanol. In the chromatogram of matrix extract, no interference is shown near the DA peak but the matrix has produced a shift in the retention time, present also in the internal standard peak. In the chromatogram of matrix extract (bottom, Figure 1), no interference is shown near the DA peak, but the matrix has produced a shift in the retention time, present also in the internal standard peak.

The linearity of the calibration curves for the quantitative determination of DA with the internal standard was evaluated using a series of standard solutions prepared in a DA-free extract by spiking different DA concentrations (0.01, 0.05, 0.1, 0.5, 1, 5, 10, 50, and 100 *μ*g L^−1^) and a constant ENK concentration of 10 *μ*g L^−1^. The linearity with *R* ^2^ > 0.99 and the equation *y* = 3.05*x* + 0.38 were obtained by considering the ratio between the concentrations of DA and ENK and the ratio between the relative areas. The instrumental precision at four concentration levels (0.01, 0.1, 1, and 10 *μ*g L^−1^) always carried out a coefficient of variation below 10%.

The analytical procedure for the determination of DA in mussel tissue was validated in terms of trueness and process yield while the method detection and quantification limits were evaluated through the estimation of a procedural blank.

We assessed the trueness and the yield by analyzing DA-free mussel tissue (20 mg) spiked with 1 *μ*g of DA and 0.2 *μ*g of internal standard ENK.

Trueness (*n* = 3), expressed as percent error, is the degree of closeness of a determined value to the known “true” value. In order to determine it with the internal standard, both DA and ENK were spiked into a DA-free tissue before the DA extraction. The percent error obtained with methanol as extraction solvent was 9%, demonstrating that ENK permits an accurate quantification of DA. The repeatability for three replicates was <5% (Table 1).

The extraction yield was calculated by spiking DA in the samples before the extraction procedure while the internal standard was added after the sample treatment and before the analysis. A value of 104 ± 8% was obtained using a DA-free matrix spiked with DA and ENK and extracted with methanol.

A good performance of this fast and simple procedural method to determine DA in the mussel tissue was also obtained using the certified reference material CRM-ASP-MUS-D, where the concentration of DA was certified to be 49 ± 3 *μ*g g^−1^. In this case, we added the internal standard before the extraction in order to determine the trueness, while ENK was spiked after the extraction procedure to estimate the extraction yield. The percent error was −2%, confirming the accuracy of this quantification method, while the extraction yield was lower (83 ± 9%) than the one reported for the sample with the spiked DA before the extraction, possibly due to the different adsorption of DA into the tissue between the two approaches. The repeatability was evaluated for three replicates, and the coefficients of variation were always below 10% (Table 1).

We quantified the method detection limit (MDL) and the method quantification limit (MQL) of the analytical procedure as three and ten times of standard deviation of the procedural blank (DA-free mussel tissue) (*n* = 3). We obtained a value of MDL of 9 ng g^−1^ and of MQL of 63 ng g^−1^. Our MDL was very similar to the value (6 ng g^−1^) obtained with the less selective fluorimetric method [9]. Our MDL value was higher than the value (0.28 ng g^−1^) reported by Regueiro et al. [15] through a one-line SPE step. We have proposed a simpler and cheaper procedure with high sensitivity and selectivity using a LC-MS/MS system. In Table 2, we reported a summary of main features (extraction, purification, and MDL) of existing methods and our simple method to better understand the main advantages of our proposed method.

Ciminiello et al. [10] proposed an analytical method without any purification step to determine DA in the mussel tissue, using HILIC coupled to tandem mass spectrometry. However, their MDL value was 63 ng g^−1^. We achieved the better detection limit because we decreased the matrix amount that influenced the signal in the ion source, and we applied a very sensitive and selective analytical method using LC-MS/MS.

The proposed method was applied to several bivalve molluscs, including* Mytilus galloprovincialis* (*n* = 6),* Venus gallina* (*n* = 4), and* Ruditapes semidecussatus* (*n* = 4). The samples were collected in the summer of 2012 in the Venice Lagoon and in the Marano Lagoon located in the Northern Adriatic Sea. During this period of monitoring, DA concentrations were always below the MDL (9 ng g^−1^).

## 4. Conclusions

We developed a very simple, fast, and cheap procedural method requiring no purification steps to determine trace concentrations of DA in mussel tissue, using hydrophilic interaction liquid chromatography coupled with a triple quadrupole. The procedure consisted in two consecutive extractions with methanol (2 × 10 mL), using a small aliquot of mussel tissue (20 mg).

Methanol permitted precipitating the proteinaceous material and reducing the extraction of interfering species, as confirmed by the qualitative and quantitative evaluations of the ME.

This is the first study to use an internal standard as quantification method to determine DA in this kind of matrix, permitting considering analyte losses during the extraction and processing of the sample and correcting the response of the mass spectrometer for random fluctuations. The method was validated through the estimation of its trueness, precision, extraction yield, and detection limit. These evaluations were done using both a DA-free matrix spiked with a known DA concentration and a reference certified material with DA adsorbed inside the mussels.

The accurate method developed in this study was characterized by a MDL of 9 ng g^−1^, the best value using LC/MS systems without clean-up step in sample preparation. This fast and accurate method can be used where the concentrations of DA are very low in order to prevent health hazard.

## Figures and Tables

**Table 1:** Validation parameters of preanalytical method developed in this work for three replicates.

Materials used for validation	Error %	Yield %	CV %
DA spiked mussels	9	104 ± 8	8
CRM-ASP-MUS-D	−2	83 ± 9	9

**Table 2:** A comparison between existing methods and the proposed method in this paper.

Instrumental method	Extraction	Clean-up	MDL	Reference
HPLC-UV	Aqueous methanol	Anionic SPE	30	[6]
CE-UV	Aqueous methanol	Anionic and cationic SPE	150	[8]
HPLC-FLD	Aqueous methanol	Anionic SPE	6	[9]
HPLC-UV	Aqueous methanol	Anion SPE	200	[13]
HPLC-MS	Aqueous methanol	Anion SPE	400	[13]
HPLC-MS/MS	Methanol/acetone	(Extraction with PLE)	200	[11]
HPLC-UV-MS/MS	Aqueous methanol	Online SPE	0.3	[15]
HPLC-MS/MS	Aqueous methanol	—	63	[10]
HPLC-MS/MS	Methanol	—	9	This paper

HPLC: high performance liquid chromatography; UV: ultraviolet detection; CE: capillary electrophoresis; FLD: fluorimetric detection; MS: mass spectrometry; MS/MS: tandem mass spectrometry; SPE: solid phase extraction; PLE: pressure liquid extraction.
